# Choroidal Metastasis of Non-Small Cell Lung Cancer That Responded to Gefitinib

**DOI:** 10.1155/2013/213124

**Published:** 2013-09-12

**Authors:** Iwao Shimomura, Yuji Tada, Gen Miura, Toshio Suzuki, Takuma Matsumura, Kenji Tsushima, Jiro Terada, Ryota Kurimoto, Emiko Sakaida, Ikuo Sekine, Yuichi Takiguchi, Shuichi Yamamoto, Koichiro Tatsumi

**Affiliations:** ^1^Department of Respirology, Graduate School of Medicine, Chiba University, 1-8-1 Inohana Chuo-ku, Chiba 260-8677, Japan; ^2^Department of Ophthalmology and Visual Science, Graduate School of Medicine, Chiba University, 1-8-1 Inohana Chuo-ku, Chiba 260-8677, Japan; ^3^Department of Medical Oncology, Graduate School of Medicine, Chiba University, 1-8-1 Inohana Chuo-ku, Chiba 260-8677, Japan

## Abstract

A 52-year-old Japanese woman presented with optical symptoms, including left-sided myodesopsia, blurred vision, narrowed visual field, and diminished visual acuity. Ocular evaluation revealed a metastatic tumor in the choroid. Further examinations identified pulmonary adenocarcinoma as the primary tumor. Because an epidermal growth factor receptor gene (*EGFR*) mutation was detected in a biopsy specimen, gefitinib treatment was initiated. Dramatic responses were obtained in the primary tumor and metastatic foci. Optical symptoms improved and remained stable for 5 months during the treatment, until relapse. This report demonstrates that gefitinib is effective for choroidal metastasis of pulmonary adenocarcinoma harboring an *EGFR* mutation.

## 1. Introduction

The choroid is the most common ocular site for metastasis due to its abundant blood supply. Only one-third of patients with choroidal metastasis have the primary tumor site identified at the time of diagnosis [1]. The two major primary sites are the breast and lung, and lung cancer accounts for approximately 30% of choroidal metastasis [2]. Visual symptoms may be the first manifestation of systemic metastases. Visual impairment includes myodesopsia, blurred vision, narrowed visual field, and lower visual acuity. These symptoms are caused not only by the mass effect of the tumor, but also by increased subretinal fluid, retinal edema, and retinal detachment. Persistent retinal detachment ultimately results in irreversible visual loss that greatly reduces the patient's quality of life (QOL).

Because two-third-of-patients with choroidal metastasis of lung cancer are benefitted by any treatment [3], early recognition and therapy are important to maximize a patient's QOL. In general, systemic chemotherapy alone is effective if the original tumor is susceptible to the cytotoxic agents. Recent progress in molecular targeted drugs has demonstrated favorable outcome for ocular tumors due to their prompt and high response rate [1, 2]. Epidermal growth factor receptor tyrosine kinase inhibitor (EGFR-TKi) is also expected to be a promising treatment for ocular metastasis of non-small cell lung cancer (NSCLC) harboring an *EGFR* mutation, although little information has been provided. 

## 2. Case Report

**Figure 1:**
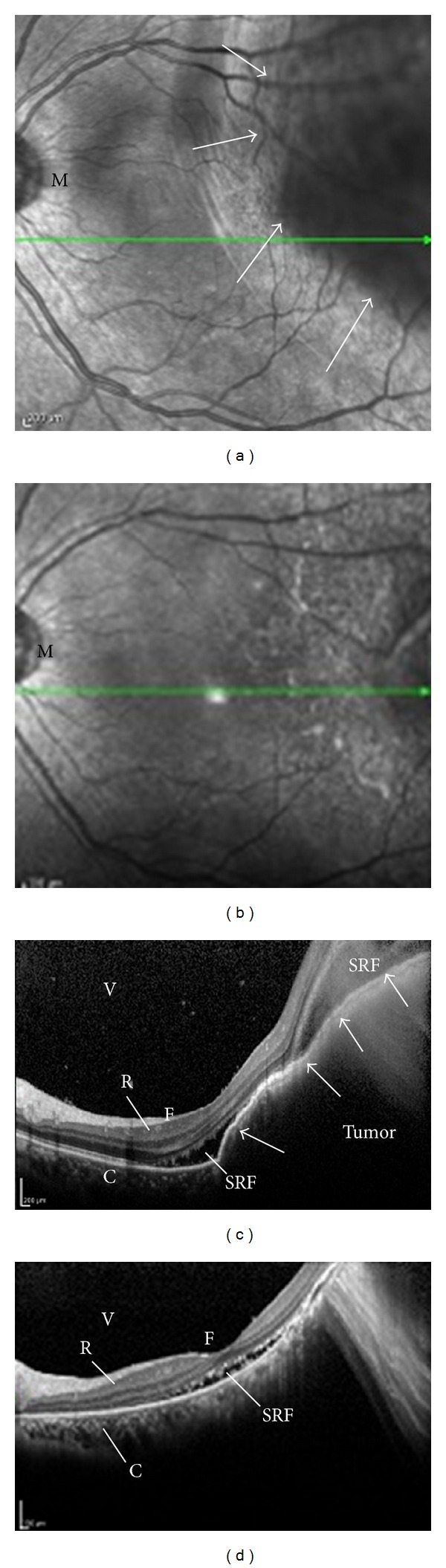
(a) Photograph of fundoscopy in the involved eye (left-side) before gefitinib treatment. A large elevated lesion in the superotemporal quadrant was found (arrows) with exudation. The patient's best corrected visual acuity in the left eye was 20/200 before treatment. (M: macula). (b) The elevated lesion disappeared a month after the onset of gefitinib treatment. Her best corrected visual acuity in the left eye improved to 20/100 after treatment. (c) Optical coherence tomography (OCT) before treatment corresponding to (a). A dome-shaped large mass with high reflectivity occupied the choroid, pushing the retina into the vitreous-cavity side (arrows). Accumulation of the subretinal fluid was also observed. (C: choroid, F: fovea centralis, R: retina, SRF: subretinal fluid, V: vitreous body). (d) Complete flattening of the tumor and decreased subretinal fluid were observed, demonstrating a dramatic response to gefitinib.

A 52-year-old Japanese woman, a current smoker, presented with optical symptoms, including left-sided myodesopsia, blurred vision, narrowed visual field, and diminished visual acuity. Because these symptoms gradually became worse, she was referred to the ophthalmologist a month after symptom onset. Ocular evaluation by fundoscopy and optical coherence tomography (OCT) revealed a solitary metastatic choroid tumor in her left eye (Figures 1(a) and 1(c)). Her best corrected visual acuity was 20/16 in the right eye and 20/200 in the left eye. For the ocular lesion, systemic chemotherapy was recommended by the ophthalmologist because the tumor size was beyond the scope of local treatment such as external-beam irradiation.

**Figure 2:**
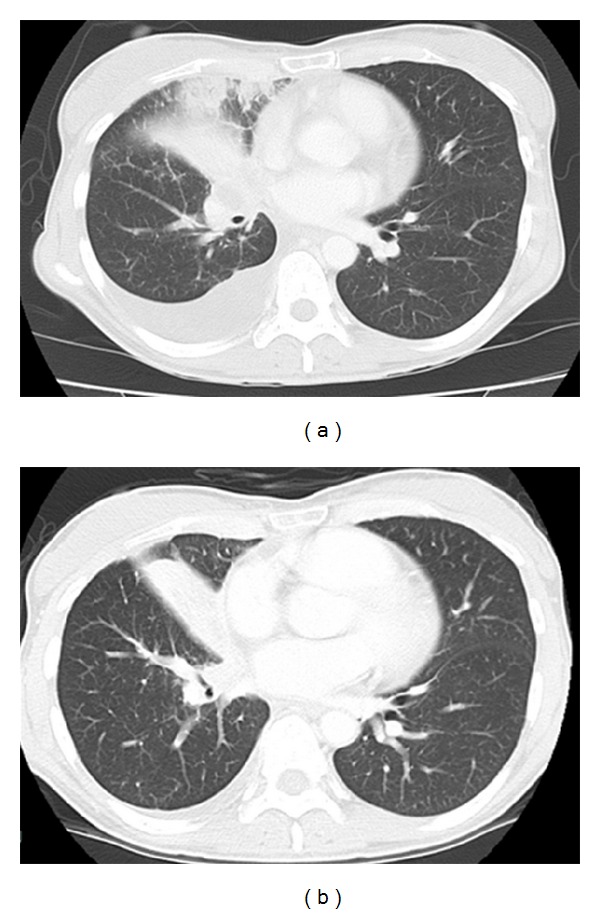
Chest computed tomography (CT) scan before treatment (a) and a month after gefitinib initiation (b). Size reduction of the primary tumor and disappearance of right-sided pleural effusion were observed.

Subsequently, a chest computed tomography (CT) scan and transbronchial biopsy (TBB) were performed to diagnose adenocarcinoma of the lung. She had malignant pleural effusion and multiple metastatic foci in bones that were asymptomatic (Figure 2(a)). The stage of her disease was determined as IV (T4N3M1b). Genetic testing of the TBB specimens identified an exon 19 deletion mutation (delE746-A750) of *EGFR*. This mutation predicts beneficial response to EGFR-TKi. She was administered oral gefitinib (250 mg daily) as the first-line treatment, which yielded dramatic responses both in the primary site and metastatic lesions within a month (Figure 2(b)). Fundoscopy (Figures 1(a) and 1(b)) and OCT (Figures 1(c) and 1(d)) revealed the almost complete disappearance of the choroidal tumor and reduction of the subretinal fluid a month after gefitinib initiation. Although the improvement of visual acuity was slight (from 20/200 to 20/100 in the left eye), myodesopsia and blurred vision dramatically improved by 3 weeks after the treatment and remained stable during the first-line therapy. Unfortunately, gefitinib was discontinued due to disease progression, with new brain metastases becoming apparent. The subsequent chemotherapy consisted of cisplatin and pemetrexed; however, these failed to improve the visual symptoms and elicited no tumor shrinkage. However, she was satisfied with the improved vision that lasted for approximately 5 months during the gefitinib treatment course, until relapse.

## 3. Discussion

In this paper, we showed the effectiveness of gefitinib for choroidal metastasis of NSCLC as the first-line treatment. To our knowledge, only three reports have previously used EGFR-TKi to treat choroidal metastasis of NSCLC [3–5]. However, two of these reports lack detailed information regarding the *EGFR* mutation, and one used erlotinib in combination with intravitreal bevacizumab. Because patients with normal EGFR gain no benefit from administering gefitinib [6], it is crucial to demonstrate the *EGFR* mutation status before choosing this treatment.

The choroid is external to the blood-retinal barrier, and systemic medication freely diffuses into the choroid via the fenestrated endothelium of choroid capillaries [5]. This is different from retinal tumors, which are protected from systemic drug exposure by the blood-retinal barrier. Systemic chemotherapy alone is thus effective for choroid metastases if the primary tumor is susceptible to anticancer agents. In NSCLC, however, systemic chemotherapy with cytotoxic drugs has drawbacks such as the time delay to the first response and limited drug efficacy. One of the merits of first-line EGFR-TKI is the prompt and dramatic response for patients with an *EGFR* mutation. We demonstrated that our patient expressed an exon 19 deletion mutation of *EGFR* in the primary tumor site and required a rapid response to avoid irreversible visual symptoms; therefore, we chose gefitinib as the first-line treatment. Indeed, almost complete remission of the choroidal lesion was achieved within a month.

For patients with NSCLC harboring EGFR mutations, complete use of EGFR-TKi is the key to success in treatment [6]. Recent studies have demonstrated that, compared with platinum doublets, first-line gefitinib prolonged progression-free survival while preserving a favorable QOL [7]. In the present case, blurred vision and narrowed visual field were promptly improved by gefitinib. Because visual impairment is a key factor that has an impact on a patient's QOL, we believe that EGFR-TKi should be initiated early in the treatment course [8]. 

Unfortunately, gefitinib was delivered for only 5 months before the disease progressed via new brain metastasis. How much benefit do patients gain by continuing EGFR-TKi beyond the disease progression is a matter that warrants further discussion. 

In conclusion, we have shown the effective use of EGFR-TKis for choroidal metastasis of NSCLC with an *EGFR* mutation. To prevent or at least delay irreversible visual loss, EGFR-TKi should be initiated earlier and should be continued as long as possible during treatment.
